# Vulnerability to Incest – Findings From a Comparative Single-Case Study of the Onset of Intrafamilial Child Sexual Abuse

**DOI:** 10.5964/sotrap.13087

**Published:** 2024-10-08

**Authors:** Judith A. Iffland, Jana Thomas

**Affiliations:** 1 Medical School Hamburg, Hamburg, Germany; 2 Private Practice, Rostock, Germany; Centre for Criminology (Kriminologische Zentralstelle – KrimZ), Wiesbaden, Germany; Simon Fraser University, Burnaby, Canada

**Keywords:** incest vulnerability, risk factors, entitlement, family systems, intrafamilial sexual abuse

## Abstract

Previous research about intrafamilial child sexual abuse was not able to identify specific risk factors that distinguish this unique subgroup from other sexual offending subgroups. In comparison to other groups of sexual offenders, men convicted of intrafamilial sexual child abuse (ICSA) are found to exhibit more similarities to non-offending fathers than extrafamilial sexual offenders. Consequently, the risk assessment of sexual recidivism among “incest offenders” lacks evidence-based evaluation criteria. Given the suggestion that family system factors should be included in research on the onset of ICSA, we employed the *Vulnerability to Incest Model* proposed by Trepper and Barrett (1989, https://doi.org/10.4324/9780203776605) in a qualitative single-case analysis. A comparative analysis of ten court evaluations of ICSA offenders revealed that all families in which ICSA has occurred demonstrated at least two vulnerability factors. The analyzed offenders exhibited comparable patterns of masculine sexual entitlement. The utility of this recently developed construct for sexual violence research is discussed and implications for further research proposed.

## Abstract (plain language summary)

**Background:**

There is less research about sexual abuse in families compared to other types of sexual offenders. Because more and more people are being convicted for child sexual exploitation material (CSEM) offenses (so-called “child pornography”), child protection agencies and family courts often ask for psychological evaluations to assess the risk of incest offending behavior by already convicted fathers who still live with their children. We do not understand incest offenses as much as we do sexual abuse by other subgroups of offenders and there is only little empirical evidence to assess the risk of incest. This makes it difficult for clinicians who work with families to know if a (step-)father is likely to abuse his children. We need more research about why individuals commit incest offenses and about the risk factors related to the family system.

**Why was this study done?:**

This qualitative study was done to understand incest offending from different points of view. Given that incest occurs within the family, the familial dynamics and spousal relationship were analyzed. The present study investigated potential reasons about why in some families abusive behaviors occur and in others not. We also aimed to find new ways to study child sexual abuse in families that could help to create effective prevention strategies.

**What did the researchers do and find?:**

We used court reports of incest offenders and analyzed them using an existing theory on familial incest vulnerability. Although incest offenders were vulnerable to sexual violence, their family and social circumstances were the main reasons for the incest. The offenders seemed to think they had the right to have sex with their family members. One reason for that could be because they had been taught about sex in a “traditional way”. We also looked at whether the abuse and neglect the offenders themselves experienced as children made them feel entitled. The construct of “masculine sexual entitlement” could be a reason for this finding. The analyzed incest offenders had a problematic relationship with the children’s mother. This may have reduced the father’s sense of entitlement regarding sexual satisfaction, dominance, and control.

**What do these findings mean?:**

The findings should encourage other researchers to look at how families and men’s attitudes towards masculinity and sexuality contribute to incest offending. However, the study sample was very small, so further investigations should examine larger samples and gather more information like, for example, about the (former) partners of incest offenders.

## Highlights

- Trepper and Barrett designed a *Vulnerability to Incest Model* barely empirically tested.
- A comparative single case analysis revealed the existence of multiple vulnerabilities to intrafamilial child sexual offenses (ICSA).
- The individual offender’s vulnerabilities are influenced by the family system.
- Masculine sexual entitlement was identified as a potential vulnerability factor for ICSA.

“*Of all the problems facing society, incest is one of the most enigmatic*.” (Trepper & Barrett, 1989, xiii)

Intrafamilial child sexual abuse (ICSA) is a serious social problem that can result in different health-related consequences for victims (Jina & Thomas, 2013; Lippus et al., 2020; Maniglio, 2009). International Studies with representative samples found prevalence rates ranging from 7.8% (Mathews et al., 2024) to 13% (Stoltenborgh et al., 2011) for different variants of sexual abuse perpetrated by an adult caregiver. The damaging effects for victims of child sexual abuse underlines the importance of early prevention strategies, especially regarding ICSA. Incest as defined by Forward (1978) can be described as “any overt sexual contact between people who are closely related or who perceive themselves to be closely related, including step-parents and step-siblings.” (as cited in Wash & Knudson-Martin, 1994). As defined by Forward (1978), an act is incestuous when it violates the special trust that exists between a parent figure or sibling. As Seto (2018) notes, incest differs from ICSA in that it can involve both coercive and noncoercive sexual interactions. In order to achieve the objectives of the present study, the article employs the term ICSA, as it focuses on coercive sexual interactions between adults and children.

To prevent children from becoming victims of ICSA, it is imperative that health professionals are enabled to accurately assess the risk of sexually abusive behavior exhibited by primary caregivers, including fathers, stepfathers, and other adults in similar roles. In light of the rapid increase in convictions for child sexual exploitation material (CSEM) offenses (Babchishin et al., 2023), institutions (e.g., those involved in child welfare) are increasingly required to assess family systems for their risk of ICSA (Garstang et al., 2023; Iffland & Schmidt, 2023; Lindenbach et al., 2022). However, the dynamics of ICSA are not nearly as well understood as other forms of sexual violence (the so-called “puzzle of incest”; Seto et al., 2015). Recent studies did not find significant risk factors in terms of sexual recidivism of individuals with a history of sexual offenses against biological or sociolegal children (Pullman et al., 2017). Since many researchers have speculated about the dark figure being especially high in ICSA (Hansmann & Eher, 2020; Pusch et al., 2021), it is possible that the published research about offender or victim characteristics may not be representative of ICSA. Eher and Ross (2006) argued that the significantly longer time periods between offending and convictions in ICSA offenders (in comparison to individuals convicted for extrafamilial child sexual abuse) demand an extension of the follow-up period in recidivism research to eight years rather than the conventional five years. They hypothesized that not the risk of re-offense, but the risk of reconviction might be lower in ICSA offenders. However, in a 10- to 15-year follow-up study conducted by Nilsson et al. (2014), the researchers also reported significantly lower sexual recidivism rates of the intrafamilial subgroup compared to individuals with history of extrafamilial child sexual abuse offenses.

Usually, the percentage of ICSA offenders in validation samples of the most commonly used standardized risk assessment tools like the Static-99 (Hanson & Thornton, 1999), Static-99R (Phenix et al., 2017), or the Stable-2007 (Hanson et al., 2007) is small. Those studies which include a substantial sample of individuals with a history of ICSA found relatively low recidivism rates between 0.9% (Rettenberger et al., 2010) and 8.7% (Hanson, 2002) for sexual recidivism. However, because recidivism rates for individuals convicted of ICSA often base on other forms of sexual offenses such as extrafamilial sexual offenses, sexual assault, or rape, the actuarial risk may not be accurate for individuals convicted solely of ICSA (Hansmann & Eher, 2020). Rice and Harris (2002) identified the Sex Offender Risk Appraisal Guide (SORAG) as a suitable instrument for the use in individuals with a history of ICSA. In their analysis of the *Violence Risk Scale: Sexual Offense Version* (VRS:SO; Olver et al., 2007), Goodman-Delahunty and O’Brien (2014) found that the Area Under the Curve (AUC) for the group of individuals convicted of ICSA did not reach statistical significance, rendering this tool unsuitable in terms of the predictive validity in this subgroup. Therefore, when assessing the risk for ICSA, evaluators lack evidence-based methods for their decisions.

## Theoretical Framework for Vulnerability to Incest

**Figure 1:**
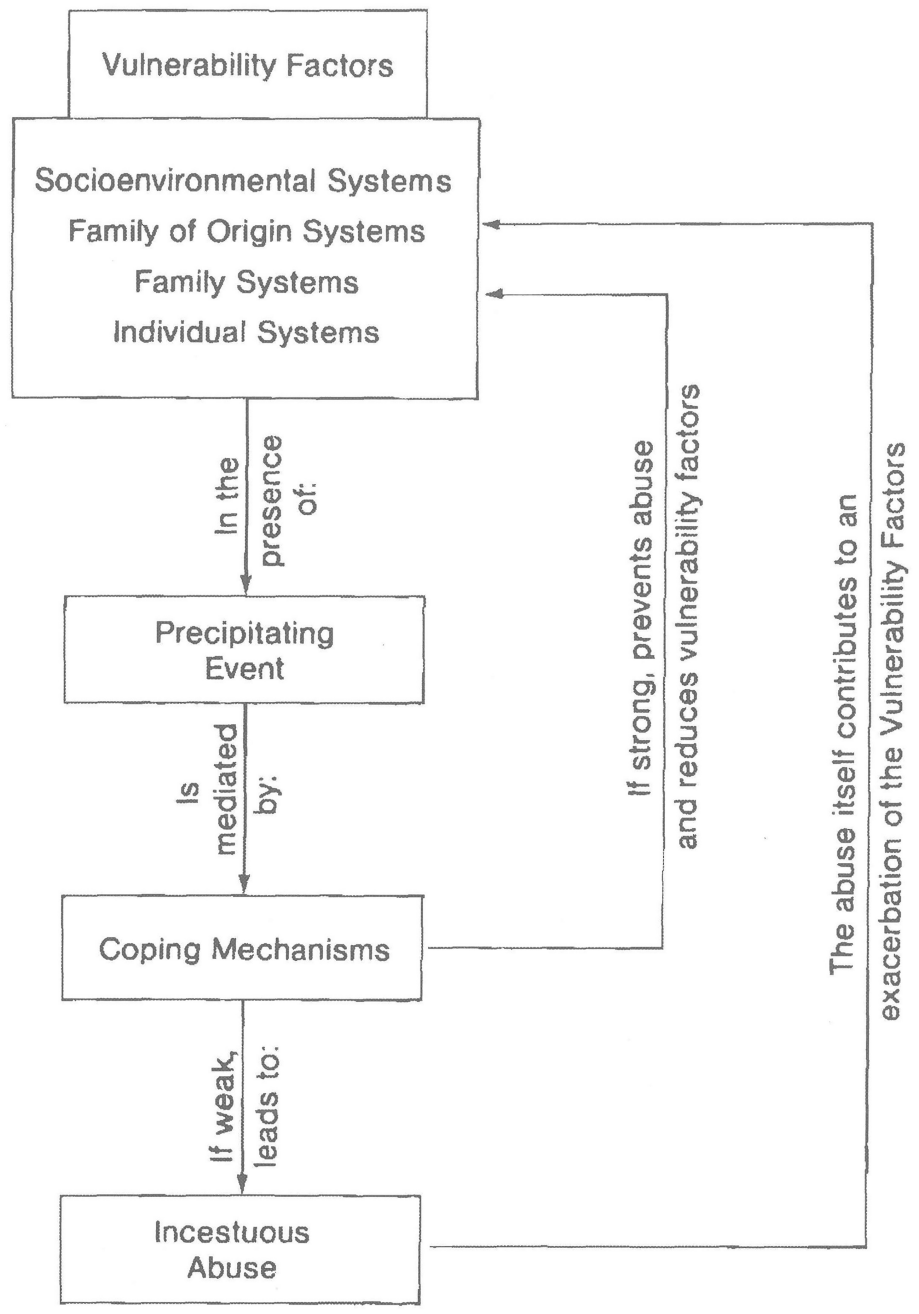
Vulnerability to Incest Model *Note.* Taken from: Systematic Treatment of Incest: A Therapeutic Handbook by T.S. Trepper & M. J. Barrett, p. 23. ©1989 by Routledge.

Different scientists discussed whether to integrate aspects of family dynamics into the research of ICSA (Greenberg et al., 2005; Kresanov et al., 2018; Martijn et al., 2020; Paquette et al., 2022; Pullman et al., 2017; Seto, 2018; Seto et al., 2015). Seto et al. (2015) summarized these dynamics in his review as (a) fathers or step-fathers assume an authoritarian, patriarchal role, (b) the marital relationship is typified by a lack of sexual intimacy and high in conflict, (c) mothers are described to be dependent on the fathers, financially or otherwise, and (d) the victimized daughters adopt the role of a surrogate partner, not merely in a sexual sense, but also with regard to intimacy and the performance of household tasks, such as the supervision and care of younger children. The *Vulnerability to Incest Model* (VTIM), as proposed by Trepper and Barrett (1989), may prove a useful framework for understanding ICSA from a systemic perspective. The authors defined “incest” as sexual contacts or behaviors initiated by an adult relative (including stepfamily members) or an adolescent or child relative. They included both stable and dynamic risk factors as well as protective factors for the onset of ICSA in their theoretical framework (see Figure 1). Trepper and Barrett (1989) asserted that there is no one particular cause of incestuous abuse, but that all families are endowed with a degree of vulnerability that may manifest as incest when a precipitating event occurs and the family’s coping skills are inadequate. They identified four major vulnerability factor areas: (1) socioenvironmental factors, (2) family-of-origin factors, (3) individual psychological factors, and (4) family systems factors. Their VTIM attempts to understand ICSA as an interaction between various external, family, and internal systems rather than focus the attention solely upon the offending (step-)father. It is important to note that the framework developed by Trepper and Barrett (1989) was originally intended for therapeutic purposes and not for risk assessment.

### Socioenvironmental Factors

Trepper and Barrett (1989) argued that culture and society contributes to the expression of specific behaviors. In the case of ICSA, the authors stated that the cultural framework surrounding male-female relationships, the messages provided about abuse and sexuality in general, and other influences such as chronic stressors or social isolation interact with one another. Typical cultural factors would be (a) family’s acceptance of male supremacy and power. The father may hold the belief that engaging in sexual activity with a female child is his “right”, and the daughter may then accept his sexually abusive behavior because his commands were regarded as a “law” within the familial structure. (b) The differential manner in which men and women traditionally display affection may also contribute to ICSA. The offending (step-)father may be inclined to sexualize relationships, perceiving sexual intimacy as a primary means of expressing affection. (c) The extent to which parents are involved in the early care and nurturing of their children is also believed to be a potential predictor for ICSA. (d) Residing in a community that implicitly accepts ICSA or other forms of child abuse (for instance certain pornography, but also social networks who implicitly approve sexual exploitation). (f) Social isolation of a family comes with the disadvantage of lacking external control and observation provided by the community. Finally, (g) chronic disease, for example in terms of economic difficulties, may result in a reduction of coping mechanisms, an increase in risk factors such as alcohol or substance abuse, and thus an increased vulnerability of the family to ICSA.

### Family-of-Origin Factors

The family of origin influences the development of the marital and parental style of both parents. Trepper and Barrett (1989) consulted research results about the perception of parental mistreatment which was found to contribute to a father’s vulnerability to ICSA. Also, the non-offending parents’ family-of-origin should be evaluated according to the VTIM with regard to potential vulnerability factors. These include stereotypical gender roles pertaining to male and female relationships, the perceived quality of the relationships between the parents and grandparents, sexual abuse in the offending parent’s own childhood, and the degree of perceived emotional neglect, abuse, or deprivation. Trepper and Barrett (1989) identified the last factor as being of central importance, as it could potentially lead to a profound attention seeking exhibited by many individuals with a history of ICSA. Correspondingly, rage, devastated self-esteem, impulsivity, and self-damaging behaviors are mentioned to be associated with emotional deprivation experienced in childhood. Consequently, the VTIM not only assesses the individual characteristics of all family members but also the interaction between the members’ individual characters and communication styles.

### Family Systems Factors

Trepper and Barrett (1989) understood ICSA as a “family experience” but emphasized at the same time that this conception should not be misunderstood as blaming the victims. By examining the structure of the family, the typical interactions and communication styles within it, the underlying intent of ICSA may be disentangled. They analyzed whether the ICSA reflects an affection-exchange, erotic-exchange, aggression-exchange, or a rage process. From a therapeutic perspective, Trepper and Barrett (1989) suggested to determine whether the ICSA had a *merging function* (one that produces closeness and contact) or a *separating function* (one that produces distance or independence). The structure of the family is evaluated in terms of interactional patterns that facilitate the integration of the abusive (step-)parent and the child victim within the same generational framework with regard to rules and boundaries. In this manner, the child is either “pulled up” to the status of an abusive parent, or the abusive parent “falls down” to the level of a child.

Trepper and Barrett (1989) identified five potential structural factors that may contribute to an increase of the family’s vulnerability to ICSA. (1) *Father-executive*: The male (step-)parent is dominant and parents the mother along with the child. The mother is dependent, passive, and relived when the child assumes parental responsibility and the role of the “wife”. The child feels the urge to “mother” the own mother and slips into the role of a female caretaker. (2) *Mother-executive*: In contrast with the father-executive structure, this structure is characterized by a dominant and powerful mother who interacts with the male parent in a manner similar to that of a child. The male father figure may exhibit behaviors that are characteristic of adolescents, such as excessive alcohol consumption or prolonged socialization with friends or colleagues. In such cases, the ICSA may initially appear to be sibling abuse. However, it can be characterized by aggression or rage, which can be understood as a “separation function”. (3) *Third-generation mother-executive, and third-generation father-executive:* This structure is a combination of the previous two and typically involves a competition between the mother and the daughter for the father’s affections. Both parents are a generation below the mother-executive and the father-executive. (4) *Chaotic*: In this structure there is no one with extended control and all family member are on the same hierarchic level. The role of the leader is subject to constant change, which results in a lack of clarity regarding one’s role within the family. (5) *Estranged father*: In cases of emotionally uninvolved (or absent) fathers the ICSA is often demanding and aggressive in nature, when the abusive father reenters temporarily. The father returns to the same generation as the daughter due to the mother’s role as an executive, which is necessitated by his emotional absence.

Trepper and Barrett (1989) chose to separate “blended families” (stepfamilies) from the aforementioned classification. Stepfamilies may appear structurally as any of the family classification but are described as particularly vulnerable for ICSA. The authors presented several potential explanations for this increase. Men with pedophilic or hebephilic sexual interests may intentionally seek out single mothers. Sexual behaviors with a stepchild may be perceived as less taboo due to a lack of cognitive dissonance. Early child care and nurturance are absent, and there are more opportunities to offend since most single mothers work full-time. Furthermore, stepfamilies have less time to establish appropriate rules and boundaries regarding proximity and the consideration of individual needs for autonomy and distance.

### Individual Personality/Psychopathology Factors

The VTIM conceptualizes individual personality and psychopathology as a self-contained system, which may contribute to a family’s vulnerability to ICSA particularly when interacting with the individual personalities of the other family members. Trepper and Barrett (1989) commented on the low consensus concerning risk factors for ICSA (even in 1989) and identified the following factors as relevant psychopathologically associated vulnerability factors: personality disorders, cognitive distortions, deviant sexual orientation, and (though very rare) psychosis. Furthermore, Trepper and Barrett (1989) emphasized the importance of assessing sexual attitudes and beliefs, sexual fantasies, general sexual knowledge, and body image in the offending (step-)father. “For example, a particular vulnerable combination might be the father’s rigid attitudes, incest fantasies, poor quality and low frequency of sexual behavior with his wife, poor body image, and little factual sexual knowledge.” (Trepper & Barrett, 1989, p. 97). Furthermore, the concept of sexual compulsivity is discussed. Regarding the nonoffending mother, it is recommended that childhood trauma, emotional absence, or incapacitation, sexual disorders such as inhibited sexual desire, role reversal with the daughter, and personality characteristics in general should be assessed. For example, a stepfather with pedophilic or hebephilic sexual interests and increased impulsivity may refrain from abusing his stepdaughter if the mother’s personality style is characterized by self-confidence, independence, and attentive protection of her child. Trepper and Barrett (1989) emphasized that even in the most vulnerable family ICSA may not occur. In accordance with their theoretical framework, the occurrence of ICSA depends on the presence of a triggering event, which they referred to as a “precipitating event”. These triggers may include alcohol and/or substance abuse, acute stress resulting from factors such as job loss or the onset of a physical disability, changes in the family structure (e.g., the birth of a child), or circumstances that present a unique opportunity for sexual abuse, such as a prolonged hospital stay of the mother. Nevertheless, even in such a triggering incident, Trepper and Barrett (1989) hypothesized that the presence of effective coping mechanisms within the family has the potential to prevent ICSA. Adequate coping mechanisms include the effective use of social networks, strong religious beliefs, therapy, and self-help groups.

### The Present Study

Since the VTIM has rarely been empirically tested, the aim of this exploratory qualitative study was to examine the VTIM based on forensic risk assessment cases. We conducted a retrospective analysis of ICSA cases with the objective of evaluating the continued relevance of this framework, which was developed nearly four decades ago, in explaining ICSA as defined by Forward (1978). Given that previous research on ICSA has been unable to find discriminate risk factors in individuals with a history of ICSA, we chose a systemic perspective using an exploratory, qualitative approach. The objective of this study was also to identify new research areas for future investigations of the enigmatic phenomenon ICSA.

## Method

We used systematic comparative analysis developed by Jüttemann (1990, 2009). With his concept of “comparative casuistry”, Jüttemann (1990, 2009) calls to study the development of certain phenomena (here: ICSA), to understand and explain them in the context of their origin and causation. Comparative casuistry aims to develop new hypotheses via single-case analyses and can be categorized as a (quasi-)experimental procedure. It is a repetitive process and an exploratory investigation of causal relationships and developments. Starting with single-case analysis, the method systematically compares phenomenological similarities and differences. The method is similar to *Grounded Theory* (Glaser & Strauss, 2017) and has been demonstrated to be useful in psychological research (Eberl et al., 2012; Habermann et al., 2008; Novak et al., 2023; Taubner et al., 2016). The aforementioned global categories of the VTIM served as a framework for the analyses, which included socioenvironmental factors, family-of-origin factors, family systems factors, and individual personality factors.

Data protection and anonymity was ensured with reference to the German law (code of criminal procedure, section 476). Informed consent of the included offenders could not be attained retrospectively due to missing contact information.

### Sample

The sample consisted of forensic court reports conducted by the authors who have an expertise in forensic risk assessment. The court reports were conducted between 2010 and 2019 and included a prison sample. All reports were subjected to retrospective analysis. The inclusion criterion was a conviction for ICSA. The exclusion criterion was the presence of sibling ICSA. The authors conducted a review of their own archive of completed risk assessment cases and identified a total of *n* = 10 court reports that met the inclusion criteria. All evaluated reports included male individuals, the mean age at the time of the initial ICSA offense was *M* = 37.7 years (*SD* = 10.2, *range* = 28–62 years). All victims were female, their age of onset of the ICSA ranged from 5 to 13 years. Eight of the ten individuals included in the study were responsible for victimizing a single child. The sample was composed of 40% biological fathers and 60% sociolegal fathers. Ninety percent of the male perpetrators engaged in sexual penetration with the victims at some point, whether vaginal, oral, or anal. Eight men used physical violence in the context of the sexual abuse, two persons used direct verbal threats. Since the court reports did not include sufficient information regarding the abused child’s mother and her family-of-origin, we had to focus primarily on the information provided by the convicted male and existing file information.

## Results

Table 1 presents the findings of the comparative case analyses regarding the global categories extracted from the VTIM. It can be seen that the included cases revealed numerous vulnerabilities as defined by Trepper and Barrett (1989). The (step-)fathers’ family-of-origin demonstrated several vulnerability factors such as out-of-home placement, different forms of abuse as well as a problematic sex education. Examples included in the variable “problematic sex education” included conservative, religious or traditional opinions regarding sex. In 40% of the sample such family-induced perceptions regarding sexuality were present. Additionally, seven out of ten cases demonstrated further difficulties in their psychosexual development. These men reported feelings of increased shame, a suppressed sexual interest, conflicted intimate relationships, or a pronounced disgust regarding oral and anal sex. The following case example underlines this finding:

Mr. S criticized his marital sexuality as “not that great”. His wife was sexually dominant, even “sexually addicted”, which made him feel disgusted.

**Table 1:** Comparative Casuistic Analyses

Themes	Examples
Socioenvironmental factors	
Partner-related issues	
History of conflicts	Separation Sudden infant death syndrome (SIDS) Unwanted pregnancy
Problems in sexuality	Missing oral sex Low frequency
Intimacy issues	Estrangement
Intimate partner violence	
Socioeconomic stress	
Unemployment Financial problems Problematic work environment	
Family-of-origin factors	
Offending fathers’ family of origin	
Out of home placement	Children’s home Foster families Raised by relatives
Experience of physical and sexual violence and neglect	Emotional absence Indifferent parental behavior Physical violence perpetrated by the mother Sexual abuse perpetrated by female guardian Witnessing sexual violence towards the mother Physical abuse perpetrated by the father Extrafamilial sex abuse
Problematic sex education	Sex as a taboo Forbidden masturbation Forbidden use of pornography Traditional views regarding premarital sex
Alcohol abuse	Mother abusing alcohol Father abusing alcohol Other relatives abusing alcohol
Family systems factors	
Hostility towards women	
Offender displays misogynic attitudes	Females against males Contemptuous remarks regarding women
Intimate partner violence (IPV)	
Traditional gender roles	
Sex belongs into marriage	
Single parent	
Individual personality factors	
Psychopathology	
Chronic substance abuse	Alcohol abuse Substance abuse (cocaine)
Pedophilic disorder	Nonexclusive pedophilic disorder
Personality disorder	Schizoid personality disorder Antisocial personality disorder Personality disorders NOS (narcissistic, dependent, compulsive, emotional-instable characteristics)
Other	Mild intellectual disability
Physical diseases	
Sexual organs	Erectile dysfunction Vasectomy
Disabilities	Morbus Scheuermann Club foot
Chronic diseases	Diabetes Epilepsy
Criminal history	
Previous sex offenses	Extrafamilial child sexual abuse
Other offenses	Theft, felony, fraud, robbery, traffic offenses, assault
Decreased social competence	
Missing social contacts Outsider Missing social integration in childhood/adolescence	
Precipitating event	
Substance abuse	
Alcohol intoxication	
Stressful working situation	
Mothers’ absence	
Short-term absence Single father	
Perception of humiliation through child´s mother	
Increasing financial dependence Dominant spouse Concealed pregnancy Infidelity	

It is noteworthy that a higher proportion of the individuals convicted for ISCA were victimized by a female guardian compared to male persons, including instances of physical abuse, sexual abuse, and emotional neglect. The ratio was determined to be 3:1, indicating that in three instances, the perpetrator of sexual victimization was female, while in one instance, the perpetrator was male. Two men reported being a victim of ICSA themselves, in both cases a female guardian was the offending person. One male reported being a victim of extrafamilial child sexual abuse. Four out of ten offenders described chronic alcohol abuse in their family-of-origin. However, there were also cases with no noticeable risk factors in the family-of-origin, as the following case example illustrates:

Mr. A described his family-of-origin as stable and caring. However, he himself developed different chronic diseases that made long hospital stays necessary. Due to his absence from school, he felt isolated, socially incompetent and envious of his healthy siblings. Sexual needs were suppressed in adolescence, Mr. A also felt incompetent within intimate relationships. His sexually experienced wife was his first sexual contact. In the abusive sexual contact with his daughter, he felt sexually experienced and secure. He stated “It [the sexual abuse] stayed within the family.”

A comparison of this case with the other included individuals with a history of ICSA revealed that 90% of the sampled men were diagnosed with at least one psychiatric disorder included in the DSM-5 or ICD-10. Five individuals were diagnosed with a chronic alcohol use disorder; however, two others also exhibited problematic alcohol consumption that did not meet all of the necessary diagnostic criteria. Four males exhibited personality disorders. Forty percent of the subjects were diagnosed with a nonexclusive pedophilic disorder. A total of 40% of the included individuals were tested with an IQ below average (< 85 points). In nine out of ten cases the *Psychopathy Checklist-Revised* (PCL-R; Hare, 2003) was rated. The mean score was *M* = 14.5 (*SD* = 9.2; *range* = 3-32), which indicates on average low to medium psychopathic traits in the sample. Seven males had a history of criminal offenses, two of these individuals had previously been convicted of child sexual abuse, which means that they have recidivated with the offense under evaluation.

Analyzing the global category *socioenvironmental factors*, we extracted two subcategories: In all families a minimum of two vulnerability factors could be identified, partner-related issues were prominent in 80% of the cases (“I was afraid of loneliness, afraid of being disappointed in another relationship.”). Eight individuals with a history of ICSA reported intimacy issues and 60% complained about the sexuality prior and/or during the sexual abuse:

“We drifted apart, had no sex. Maybe she [child’s mother] had an affair, I don’t know. Everything was too much, why did I have to do everything? Nobody listened to me!”

In three cases, a history of negative partner-related incidents was also extracted, including unwanted pregnancy, sudden infant death syndrome (SIDS), and a period of separation. Furthermore, four out of ten men reported instances of infidelity of the children’s mother. Additionally, in three families intimate partner violence (IPV) was perpetrated by the offending (step-)father. Due to missing information included in the archived court reports, only two subcategories emerged in the global category of *family systems* vulnerabilities: hostility towards women and traditional gender roles, which could be identified in three out of ten cases.

Mr. S stated: “I hate women, especially those who lie and cheat. My Ex used to cheat, she lied about cheating on me. But she did!”

One individual stated: “I had the feeling, in our family we stood women against men.” He thus included the victimized daughter into the category “women”, implicitly pulling her up into the parents’ generation in accordance with Trepper and Barrett (1989). The same individual described his relationship with the victimized daughter as follows: “From my point of view, we were on equal terms.” Accordingly, a comparison of the cases revealed that in five out of ten cases the victimized daughter was perceived as a substitute spouse.

Mr. S. explained his abusive behavior toward his daughter by looking for a substitute because his wife was cheating on him. He chose his daughter as a substitute because she was “there”.

The following case example illustrates how the different systems in the VTIM could be linked:

Mr. B. was sexually abused by a female guardian in childhood. In adolescence, he claimed to be “the man in the house”, tried to dominate and suppress his younger sister and developed a misogynistic attitude. His psychosexual development was perceived as shameful, intimate relationships with women were sought, but own sexual wishes not communicated. Female partners were described as self-confident and in control, while Mr. A was sexually aroused by feelings of dominance in the sexual interaction. When his spouse concealed her pregnancy with their daughter, he felt humiliated. In the following years bringing up the child, the spousal relationship was lacking intimacy and sexual contact. When the daughter was prepubescent, the incestuous abuse began with increased violence and penetration.

When systematically comparing the family structure at the time of onset, all but the father-executive models as defined by Trepper and Barrett (1989) could be found (merging and separating function; mother executive etc.), but none was prominent.

## Discussion

The aim of this study was to qualitatively analyze existing material of ICSA cases in terms of the theoretical fit with the VTIM developed by Trepper and Barrett (1989). Although their model classified the systems of equal importance, due to the information available in the reports we focused mainly on vulnerabilities regarding the offending (step-)fathers. A number of individual risk factors could be identified from the data, including substance abuse and personality disorders. These risk factors have been identified in numerous studies as prevalent among individuals with a history of ICSA (Biedermann et al., 2023; Eher et al., 2019), and they are significantly correlated with sexual recidivism (Hanson & Morton-Bourgon, 2005, 2009). The only study known to the authors which empirically investigated the VTIM was conducted by Terry Trepper himself (Trepper et al., 1997). This study used therapists’ evaluations of families in which ICSA has occurred and found that pedophilic disorders were not frequent in individuals with a history of ICSA. This finding aligns with previous research comparing individuals with a history of intrafamilial and extrafamilial sexual abuse (Marshall et al., 1986; Martijn et al., 2020; Schmidt et al., 2013; Seto, 2018). However, by using phallometric assessment Rice and Harris (2002) found that individuals with a history of ICSA were sexually deviant as a group, although not as sexually deviant as extrafamilial offenders. Pezzoli et al. (2022) employed viewing time measures with child and adult stimuli to identify community men with a propensity for ICSA. Their findings indicated that the sample of community men with a self-reported history of ICSA did not exhibit a significant pedo-/hebephilic preference. Although the sample size was limited, Pezzoli et al. (2022) referred to Seto (2018) suggesting that the sexual arousal for child stimuli is not significantly stronger than for adult stimuli. In our sample, we also found 40% to be diagnosed with a pedophilic disorder, though not exclusively. Consequently, it can be postulated that although a certain preference for pedophilic-/hebephilic stimuli is present among this group of individuals with a history of ICSA, which made them vulnerable to incestuous abuse, the sexual preference for adult stimuli remained stronger. This brings us back to the discussion about further factors, which could increase the vulnerability of ICSA.

Trepper et al. (1997) identified substance abuse as a significant vulnerability factor and a clear precipitant, which corresponds with our findings. The significance of alcohol abuse in individuals with a history of ICSA has been previously documented by other researchers, including Cole (1992) and Groth (1982), but was also identified in individuals with a history of extrafamilial child abuse (Rice & Harris, 2002). We also extracted a variety of victimization experiences in the offender’s own childhood ranging from emotional neglect to sexual abuse. Based on their findings examining the marital relationships of individuals with a history of ICSA, Lang et al. (1990) hypothesized that the lack of intimacy skills in incestuous fathers may have its origins in the men’s childhood, when they felt isolated, unloved, or deprived of bonds within their own family of origin. Martijn et al. (2020) also identified a high prevalence of childhood maltreatment history in male adolescents who had sexually offended against intrafamilial victims. Problematic experiences like paternal rejection and powerlessness were also reported earlier by Williams and Finkelhor (1990) in their review on incestuous fathers. However, Parker and Parker (1986) did not find significant differences regarding early family instability comparing individuals with a history of ICSA to nonsexual offenders. Rice and Harris (2002) identified less serious and disturbed backgrounds in individuals with a history of ICSA compared to extrafamilial child sexual abuse. Thus, our findings may not be specific to individuals with a history of ICSA. In our sample, we observed a higher proportion of child maltreatment perpetrated by female guardians, which may be associated with the subcategory “hostility towards women.” However, this may also have had a negative impact on the development of a secure attachment style. Similarly, McKillop et al. (2012) found that individuals who committed familial-onset offenses exhibited higher levels of insecure maternal attachment compared to those who perpetrated extrafamilial child abuse. However, this difference was not statistically significant.

Although we could extract certain similarities in the familial systems according to Trepper and Barrett (1989), the included cases demonstrated highly individual dynamics that contributed to the onset of ICSA. However, when comparing the extracted subcategories and themes such as traditional norms, including sexuality and gender roles, difficulties in psychosexual development, hostile attitudes towards women, or perceived humiliation by the intimate partners it could be hypothesized that the intrafamilial sexual abuse is associated with an additional global risk factor category: dysfunctional masculine gender identity. Gender identity refers to the way in which individuals perceives themselves in relation to masculine and feminine norms. These deeply ingrained images not only define attitudes, expectations, and ideals in terms of one’s own behavior, but also influence how individuals perceive others (Wash & Knudson-Martin, 1994). Traditional scripts of masculinity suggest that men should be powerful, in control, and sexually omnipotent (Person, 1993). These ideals are usually learned in the families of origin. These patterns and interactions are frequently replicated in the men’s own families, influencing their self-perception as a partner and parent (Wash & Knudson-Martin, 1994).

A recent qualitative analysis of sibling sexual abuse found that traditional gender norms and sexism were frequently prevalent in the familial environments affected by the abuse (McCartan et al., 2023). Partner-related issues such as intimacy problems, dissatisfaction with the relationship sexuality, or spousal infidelity may also be related to the construct of male sexual entitlement. Major (1987) defined entitlement as “deservingness; the person who feels entitled to a particular outcome or level of outcomes feels that he or she should receive that outcome” (p. 131). Sexual entitlement and sexist attitudes were previously identified by Hanson et al. (1994) as being associated with ICSA. They hypothesized that sexual entitlement may be the underlying link between sexual abuse and individual differences in sexually specific sexist attitudes. Drawing from our findings, male sexual entitlement may also be the link between ICSA and a pedo-/hebephilic preference. Other studies have also identified male sexual entitlement to be an important factor in understanding sexual violence (Jewkes et al., 2011; Zietz & Das, 2018). Recently, Raines et al. (2023) defined “masculine sexual entitlement” as “personal and collective attitudes, norms, and behaviors involving an exaggerated belief in masculine individuals’ right to or deservingness of sex and upholding or reinforcing patterns that contribute to these dynamics” (p. 2). Raines et al. (2023) included prioritizing own sexual needs, objectification of others and misogyny, gender essentialism, and sexual deception in the construct.

Masculine entitlement has previously been linked to ICSA offenses by Wash and Knudson-Martin (1994). The authors hypothesized that ICSA can be interpreted as a consequence of masculine entitlement resulting from a masculine gender construction and family experiences that have let to conflicts in the intimate relationship. In their *Conceptual Model of the Experience of Incestuous Fathers* (Wash & Knudson-Martin, 1994), they placed entitlement at the center of their model, which produces ICSA when additional factors of control needs and/or intimacy needs are present. Adapting their model to our sample seems to be promising: Wash and Knudson-Martin (1994) argued that a destructive sense of entitlement is related to a lack of care in previous relationships and that the individuals with a history of ICSA use incestuous abuse to get what they were deprived of earlier in life, sexually and/or emotionally. Our findings support this hypothesis: Most individuals reported several victimizations in their family of origin. Furthermore, their psychosexual development often included experiences of narcissistic insults in former intimate relationships with women. Similarly, the ICSA occurred in a familial context, with the men reporting feelings of inadequacy with regard to their gender role, at least in the majority of cases.

Wash and Knudson-Martin’s (1994) definition of *Build-up Failure* includes a conflict between the individuals’ image of masculinity and their own struggles to live up to their expectations (similar to the concept of “discrepancy strain”, see Levant & Richmond, 2016). Similarly, in our qualitative data, the CSA occurred in a familial context, wherein the male participants expressed feelings of inadequacy with respect to their conventional gender roles. Wash and Knudson-Martin (1994) additionally posited that ICSA may serve to address further shortcomings based on perceived entitlement. As a result of their perception of entitlement to love and intimacy, coupled with feelings of sexual unworthiness and vulnerability within the familial structure, sexual intimacy is employed as a means of attaining proximity and connection. Additionally, the conflict between a desire for power and control and the experience of powerlessness within the familial system and in relationships with female partners might result in the use of incestuous contact as a means of reestablishing this sense of entitlement (Wash & Knudson-Martin, 1994). In our sample, we identified similar offense characteristics and motivations that aimed to increase a sense of power and dominance. However, these characteristics and motivations are not exclusive to ICSA offenders, they were also identified in numerous studies on individuals with a history of sexual offenses (e.g., Chan et al., 2019; Cole, 1992; Groth et al., 1977; Iffland et al., 2016; Kamphuis et al., 2005).

In correspondence with Wash and Knudson-Martin (1994), this need for dominance is accompanied by a need for closeness and gratification of sexual potency that appears to have developed within the family system, particularly within the context of the intimate relationship with the children’s mother. This corresponds with Cole (1992) review of ICSA perpetrators and their familial structures. Nevertheless, the extracted subcategories in our analysis indicate the presence of another vulnerability factor not specifically included in Wash and Knudson-Martin’s (1994) model but mentioned by Trepper and Barrett (1989), which was impulsivity. Impulsivity can be described as the failure to resist a drive or impulse without considering potentially negative outcomes (Moeller et al., 2001). One of the frequently discussed behavioral subcomponents of impulsivity is response inhibition. The category “Psychopathology” extracted from the single-case analysis included Cluster-B personality disorders as well as intellectual impairments and chronic alcohol abuse which have been linked to an increased degree of impulsivity and a decreased inhibition.

In the context of child sexual offending, Turner et al. (2018) suggested that impairments in response inhibition could lead to an inability to inhibit the impulse to have sexual contact with a child. Therefore, impulsive decision making could represent a tendency to satisfy a (step-)father’s sexual desires by abusing their children, while neglecting the negative long-term consequences, such as divorce and incarceration. Turner et al. (2018) found impairments in response inhibition in individuals with a history of child sexual abuse and discussed that in real-life situations, individuals with a history of child sexual abuse may not be more impulsive than other individuals, while the presence of sexual cues (e.g., children) increases sexual arousal, which could then lead to decreased self-control skills, including deficits in response inhibition. This, in turn, can result in an inability to inhibit the impulse to sexually abuse a child. This is consistent with our findings, which did not indicate that impulsivity is a stable personality trait associated with antisociality or psychopathy in our sample. Consequently, although impulsivity is a significant risk factor for sexual recidivism (Hanson & Morton-Bourgon, 2005; Mann et al., 2010; Olver & Eher, 2020), further evaluation is required to determine the relevance of different behavioral components of the multidimensional construct of impulsivity in the context of ICSA.

**Figure 2:**
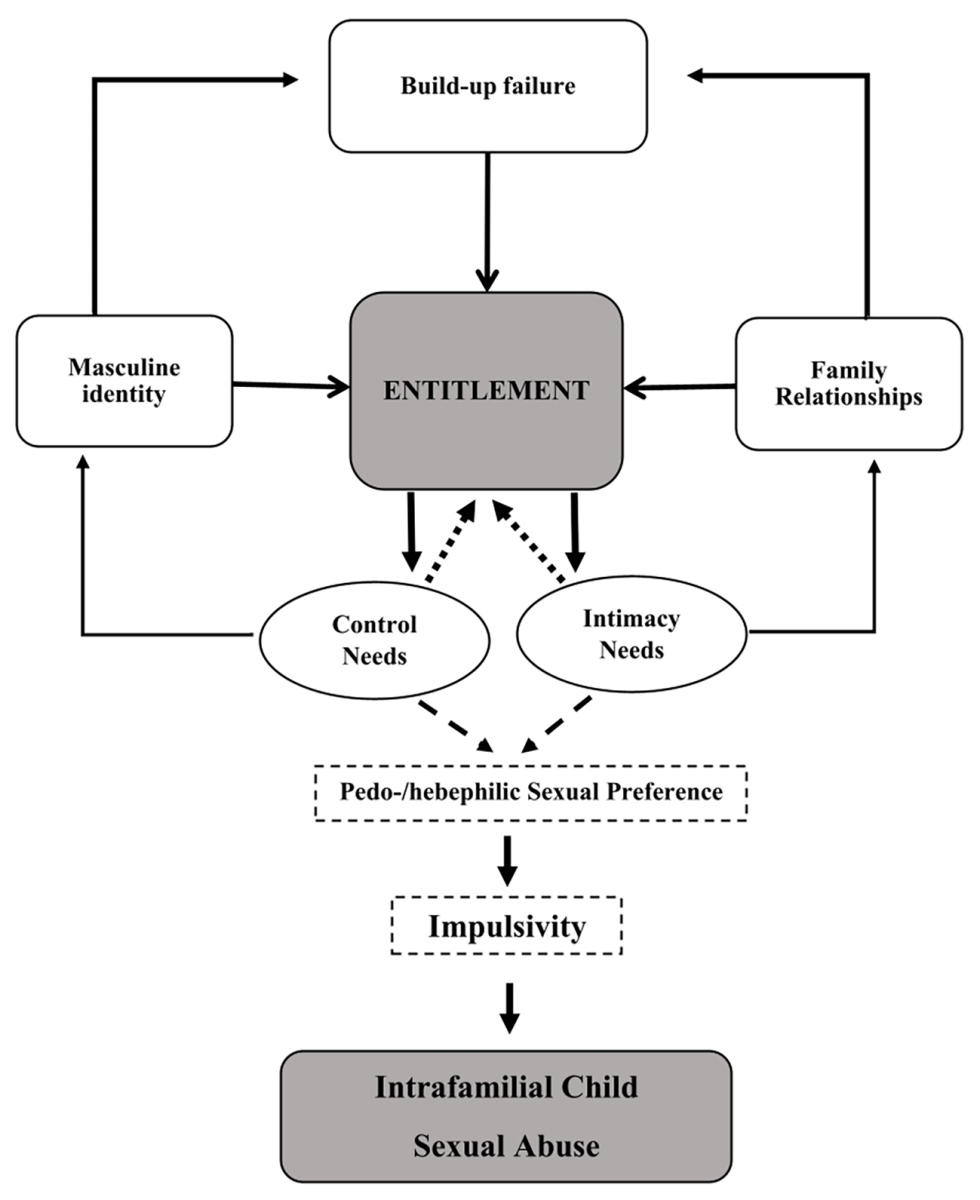
Adapted Conceptual Model of the Experience of Incestuous Fathers

In the family system, the modus operandi frequently involves strategic elements, including the engagement in sexually inappropriate behavior when the situation is optimal (e.g., when the mother is absent) or even the active creation of circumstances that facilitate sexual abuse. However, findings reported by Turner et al. (2018) suggested that impulsivity may be the precipitating factor connecting masculine sexual entitlement and the vulnerability factor of a pedo-/hebephilic sexual preference, as discussed above, with the final onset of ICSA. The development of the pedo-/hebephilic arousal may be reinforced by control and intimacy needs associated with entitlement. Therefore, it is proposed that impulsivity and pedo-/hebephilic preference be included as contributing factors to the Wash and Knudson-Martins Model (1994; Figure 2: *Adapted Conceptual Model of the Experience of Incestuous Fathers*).

### Limitations

It is important to note that this study is subject to several limitations that should be taken into account when interpreting the results. The study is qualitative and exploratory in nature. Nevertheless, we guarantee the quality of the data collected in accordance with the *Standards for Reporting Qualitative Research* (SRQR; O’Brien et al., 2014) and in terms of trustworthiness as defined by Lincoln and Guba (1985). The sample size is small and the findings are not representative of the larger population. Additionally, a comparison group, such as individuals with a history of extrafamilial sexual abuse, was not included in the study. As the present study’s sample exclusively comprises male individuals with a history of ICSA, it offers no insight into the characteristics and behaviors of female perpetrators of ICSA. Furthermore, the sample exclusively comprised offenders who had victimized females. Consequently, the findings and subsequent discussion may be constrained to male-to-female ICSA. The sampled men were not interviewed again; rather, our own court reports and only the information included was used for the comparative casuistry. Despite the fact that the reports were conducted by experienced legal psychologists who employed evidence-based assessment and evaluation criteria, several potential vulnerability factors could not be assessed, particular in relation to the abused children’s mother and the family dynamics. In a more comprehensive study by Trepper et al. (1997), it was found that approximately one-third of the families exhibited a high degree of emotional intimacy, accompanied by a demand for unwavering loyalty from family members. This was related to a reduction of personal and private boundaries as well as negative communication styles.

As in most risk assessment evaluations, the offender himself is the primary source of information. Consequently, our data is biased with regard to the information he chose to reveal and his retrospective interpretations. For example, it could not be verified whether the intimate relationships with the children’s mother were indeed conflicted and estranged, or whether the offender had reported such issues as a means of justifying or excusing his abusing behavior. However, in the Trepper et al. (1997) study, the nonoffending partners of the ICSA offenders rated their sexual satisfaction as analogous unhappy to the offending (step-)fathers’ ratings. In a previous qualitative interview study of “sex offender couples” conducted by Iffland et al. (2016), female partners of men with a history of child sexual abuse described their partner’s need for dominance and control in the relationship. This may support our hypothesis that masculine entitlement issues may act as a precipitating factor in the first onset of incestuous behavior.

Trepper and Barrett (1989) definition of a “father-executive” that is characterized by a dependent and passive mother could not be extracted from the existing files. However, this risk factor for ICSA is frequently discussed in the literature (e.g., Gul et al., 2020; Pusch et al., 2021; Seto et al., 2015; Slavič, 2020). It should be noted, however, that the included individuals with a history of ICSA were all charged and convicted, which resulted in the separation of the abused children’s mothers from them. It is possible that the father-executive dynamic is more prevalent in constellations of hidden ICSA in which the abused child’s mother is silent, contributes to, or even participates in the abuse (Schröder et al., 2023). Nevertheless, in Trepper et al. (1997) the investigation of the VTIM also revealed that less than one-third of the included families could be classified as “father-executive”. Therefore, maybe it is time for researchers to reconsider the assumption that ICSA occurs exclusively in families where mothers are submissive and unable to react. However, our findings and those of previous researchers do support the significance of the mother of the victimized child with regard to her role within in the vulnerable family structure.

### Conclusion and Implications

The findings of this qualitative study provide insight into the family systems of individuals with a history of ICSA. The VTIM enabled us to endorse their perspective on the complexity of the ICSA phenomenon. Our findings indicate that the spousal relationship with the children’s mother, particularly in regard to its impact on the offending (step-)fathers’ entitlement issues, is essential to understand the enigma of incest. The theme of masculine sexual entitlement was discussed as a central vulnerability factor, thereby reinforcing the conceptual model proposed by Wash and Knudson-Martin (1994). Aspects of masculine sexual entitlement have been previously linked to sexual aggression (see Bouffard, 2010; Hanson et al., 1994; Hill & Fischer, 2001; Raines et al., 2023). Further investigation of this association is recommended, particularly with a larger forensic sample of individuals with a history of ICSA, but also with minor-attracted persons who have not offended, in order to investigate the proposed association between entitlement and pedo-/hebephilic preference and impulsivity (e.g., Beier et al., 2015; Osterheider et al., 2011). The findings of this study may serve as a preliminary step to identify families with an increased vulnerability for ICSA. Further insight into the phenomenon of ICSA is highly relevant for the development of effective early prevention strategies and for the assessment of recidivism risk. Our findings indicate that evaluating family of origin factors, masculine sexual entitlement, marital sexual satisfaction, and its impact on the spousal relationship could contribute to the standard assessment of individual evidence-based risk factors such as personality disorders, substance abuse, sexual preference or impulsivity.

As the reader may have noticed, the majority of studies on ICSA are outdated. The most prominent era of ICSA research was more than 30 years ago. This indicates that identifying (step-)fathers with a high risk for sexually abusive behavior in terms of recidivism or vulnerabilities for onset has become a rather neglected field of research. Nevertheless, including evidence-based risk factors is of high importance in both risk assessment regarding sexual recidivism (Hanson & Morton-Bourgon, 2009) and therapeutic planning using the RNR model (“Risk/Need/Responsivity”; Bonta & Andrews, 2024). Given that research focusing on specific characteristics of individuals with a history of ICSA has indicated that they are more similar to nonoffending fathers than to other groups of sexual offenders (Pezzoli et al., 2022), it would be beneficial for future studies to include system-related risk factors in their research.

## Data Availability

The qualitative data cannot be shared due to the sensitive nature of our material and reasons of confidentiality. The coding scheme can be made available upon request by the first author.

## Social Media Accounts

@JudithIffland
